# Hepatitis B Surface Antigen Concentrations in Patients with HIV/HBV Co-Infection

**DOI:** 10.1371/journal.pone.0043143

**Published:** 2012-08-15

**Authors:** Jerzy Jaroszewicz, Thomas Reiberger, Dirk Meyer-Olson, Stefan Mauss, Martin Vogel, Patrick Ingiliz, Berit Anna Payer, Matthias Stoll, Michael P. Manns, Reinhold E. Schmidt, Robert Flisiak, Heiner Wedemeyer, Markus Peck-Radosavljevic, Jürgen Rockstroh, Markus Cornberg

**Affiliations:** 1 Department of Gastroenterology, Hepatology and Endocrinology, Hannover Medical School, Hannover, Germany; 2 Division of Gastroenterology and Hepatology, Department of Internal Medicine III, Medical University Vienna, Vienna, Austria; 3 Department of Clinical Immunology and Rheumatology, Hannover Medical School, Hannover, Germany; 4 Center for HIV and Hepatogastroenterology, Düsseldorf, Germany; 5 Department of Internal Medicine I, University of Bonn, Bonn, Germany; 6 Medical Center for Infectious Diseases (MIB), Berlin, Germany; 7 Department of Infectious Diseases and Hepatology, Medical University of Bialystok, Bialystok, Poland; 8 German Center for Infectious Disease Research (DZIF); The University of Hong Kong, Hong Kong

## Abstract

HBsAg clearance is associated with clinical cure of chronic hepatitis B virus (HBV) infection. Quantification of HBsAg may help to predict HBsAg clearance during the natural course of HBV infection and during antiviral therapy. Most studies investigating quantitative HBsAg were performed in HBV mono-infected patients. However, the immune status is considered to be important for HBsAg decline and subsequent HBsAg loss. HIV co-infection unfavorably influences the course of chronic hepatitis B. In this cross-sectional study we investigated quantitative HBsAg in 173 HBV/HIV co-infected patients from 6 centers and evaluated the importance of immunodeficiency and antiretroviral therapy. We also compared 46 untreated HIV/HBV infected patients with 46 well-matched HBV mono-infected patients. HBsAg levels correlated with CD4 T-cell count and were higher in patients with more advanced HIV CDC stage. Patients on combination antiretroviral therapy (cART) including nucleos(t)ide analogues active against HBV demonstrated significant lower HBsAg levels compared to untreated patients. Importantly, HBsAg levels were significantly lower in patients who had a stronger increase between nadir CD4 and current CD4 T-cell count during cART. Untreated HIV/HBV patients demonstrated higher HBsAg levels than HBV mono-infected patients despite similar HBV DNA levels. In conclusion, HBsAg decline is dependent on an effective immune status. Restoration of CD4 T-cells during treatment with cART including nucleos(t)ide analogues seems to be important for HBsAg decrease and subsequent HBsAg loss.

## Introduction

Chronic Hepatitis B virus (HBV) infection affects more than 350 million people worldwide. HBsAg clearance and anti-HBs seroconversion is associated with the best outcome in patients with HBV infection [1], [2]. In the natural course of HBV infection and during antiviral therapy quantification of HBsAg is a useful tool to predict the chances for HBsAg clearance [3]. The decline of HBsAg concentrations may correlate with an effective immune response against HBV and is less dependent on direct viral suppression with HBV polymerase inhibitors. This statement is supported by data that interferon alpha induced stronger HBsAg decline compared to NA therapy [4], [5]. Moreover, in a recent study HBsAg decline during NA therapy was correlated with high baseline interferon-inducible protein-10 (IP-10) levels [6], reflecting increased activity of the endogenous interferon pathway. Highest HBsAg levels are observed in immunotolerant HBeAg positive patients while lowest are detected in HBsAg carriers [7]–[9]. The link of the immune status and HBsAg concentrations may be of even greater clinical relevance in patients co-infected with the human immunodeficiency virus (HIV). The prevalence of chronic hepatitis B virus (HBV) infection in patients with HIV infection is 6–14% due to the shared routes of transmission [10]. The natural course of HBV infection can be more severe and progression of HBV related liver cirrhosis is faster in HIV positive patients [11]. Several nucleoside and nucleotide analogues (NA), inhibitors of the HIV reverse transcriptase, are also effective against HBV. One of the most frequently used NA as backbone of combined antiretroviral therapy (cART) against HIV is tenofovir, which is highly effective against HBV [12], [13]. It has been documented that long-term therapy with tenofovir reduces the risk of liver cirrhosis in HIV/HBV co-infected patients [14]. Still, HBsAg clearance and subsequent seroconversion, which represents the ultimate goal of HBV treatment, is rare. A recent study in HIV/HBV co-infected patients documented a yearly HBsAg seroconversion rate of 2.6% [15]. Monitoring of HBsAg concentrations has so far mainly been studied in HBV mono-infected patients. Two recent studies have investigated HBsAg concentrations in HIV/HBV co-infected patients [16], [17]. Both studies documented that HBsAg levels decreased only slowly despite complete suppression of HBV DNA replication during tenofovir treatment similar to reports in mono-infected patients [3]. The smaller study including 33 patients showed that HBsAg decline was not associated with HIV parameters such as HIV RNA or CD4 T-cell count. [16]. In contrast, the larger study from France including 143 patients demonstrated that HBsAg decline during TDF therapy was influenced by HIV induced immunodeficiency [17]. In HBeAg positive patients, HBsAg decline was significantly slower in patients with CD4 T-cell count <350/mm^3^. This study suggests that the immune status is relevant for HBsAg decline but the body of evidence is limited.

Here we aimed to investigate the correlation of quantitative HBsAg and HIV parameters in 173 HIV/HBV co-infected patients in a cross-sectional study. Our data provide further evidence that the immune status is important for HBsAg decline.

## Patients and Methods

### Patients and Study Design

Two hundred and fifteen HIV/HBV co-infected patients were recruited in this multicenter, cross-sectional retrospective study in 6 centers in Germany, Austria and Poland between 2001–2011. In all subjects HIV infection (positive screening and confirmatory serological tests) and chronic hepatitis B virus infection (positive HBsAg for at least 6 months) have been documented. Participating centers included Vienna (n = 72), Hannover (n = 42), Duesseldorf (n = 36), Bonn (n = 26), Berlin (n = 21) and Bialystok (n = 8). Out of 215 patients, 42 had concomitant HCV and/or HDV infection. These patients were excluded from the analysis; the final cohort consisted of 173 HIV/HBV co-infected patients. There were 148 male (85%) patients with a median age of 40 (range 17–76 yrs), 123 (71%) were receiving combination antiretroviral therapy (cART) active against HBV for a median duration of 47 (7–95) months. Predominant route of infection was MSM (n = 93, 54%), followed by IVDU (n = 20, 12%) and heterosexual transmission (n = 14, 7%), while in 46 (n = 27%) the mode of infection was unclear or endemic. The detailed characteristics of the study cohort are given in Table 1. HIV/HBV co-infected patients not receiving cART (n = 46) were compared with HBV mono-infected subjects (n = 46) without history of anti-HBV therapy. Four untreated HIV/HBV co-infected patients could not be matched due to low HBV DNA and HBeAg positivity. The control group of HBV mono-infected patients was selected from a well-defined cohort [7] and was matched for age, gender, HBeAg status and HBV DNA concentration (Table 2), which are the most relevant factors associated with HBsAg levels [3], [7]. We analyzed HBsAg levels and HIV parameters at one time-point. In addition, nadir CD4 T-cell count was recorded.

**Table 1:** Characteristics of HIV/HBV co-infected patients.

	Complete cohort(n = 173)	HBeAg(+)(n = 103)	HBeAg(−)(n = 70)
Age, years (median, 10–90% CI)	40 (27–53)	50 (25–54)	41 (28–52)
Gender, n (M/F)	148/25	93/10	55/15
cART (n, %)	123 (72)	72 (70)	51 (73)
cART duration, months (median, 10–90% CI)	47 (7–95)	49 (12–96)	46 (5–94)
Anti-HBV compound of cART:			
TDF+FTC (n,%)	71	44	27
LAM (n, %)	20	11	9
TDF+LAM (n, %)	18	10	8
TDF (n, %)	11	6	5
LAM+FTC (n, %)	3	1	2
CDC class:			
A (n,%)	60	29	31
B (n,%)	45	26	19
C (n, %)	49	36	13
Unknown (n, %)	19	12	7
HBeAg(+), n (%)	103 (59)	103 (100)	0 (0)
HBV DNA, log_10_ IU/mL (median, 10–90% CI)	1.30 (0.77–7.22)	1.94 (0.77–7.74)	1.30 (0.77–3.24)
HIV RNA, log10 copies/mL (median, 10–90% CI)	1.69 (1.60–4.49)	1.69 (1.60–5.12)	1.69 (1.60–4.87)
CD4 count, cells/µL (median, 10–90% CI)	373 (120–734)	355 (87–664)	417 (177–742)
Nadir CD4 count, cells/µL (median, 10–90% CI)	161 (16–409)	148 (11–393)	173 (23–420)
ΔCD4 count, cells/µL (median, 10–90% CI)	166 (0–501)	158 (0–431)	182 (0–518)
CD8 count, cells/µL (median, 10–90% CI)	845 (432–1691)	805 (406–1660)	915 (447–1811)
ALT, IU/mL (median, 10–90% CI)	33 (16–97)	34 (17–98)	30 (16–87)
AST, IU/mL (median, 10–90% CI)	34 (22–93)	37 (19–91)	32 (24–96)
INR (median, 10–90% CI)	1.00 (1.00–1.12)	1.05 (1.00–1.22)	1.00 (1.00–1.10)
Platelets, 10^3^/L (median, 10–90% CI)	199 (117–291)	191 (111–289)	207 (117–315)

**Table 2:** Characteristics of 46 HIV/HBV co-infected patients and 46 matched HBV-mono-infection patients not receiving anti-HBV therapy. Matched for age, sex and HBeAg status.

	HBV/HIV co-infection(n = 46)	HBV mono-infection(n = 46)	P-value
Age, years (median, 10–90% CI)	34 (23–51)	34 (22–54)	0.93
Gender, n (M/F)	40/6	40/6	1.00
HBV DNA, log_10_ IU/mL (median, 10–90% CI)	4.82 (1.07–8.04)	5.48 (2.27–8.06)	0.64
HBeAg(+), n (%)	26 (56)	26 (56)	1.00
ALT, IU/mL (median, 10–90% CI)	40 (23–228)	69 (18–207)	0.54
AST, IU/mL (median, 10–90% CI)	46 (30–131)	46 (24–149)	0.34
INR (median, 10–90% CI)	1.08 (0.90–1.50)	1.08 (0.95–1.45)	0.75
Platelets, 10^3^/L (median, 10–90% CI)	175 (124–289)	213 (106–291)	0.22
HBsAg, log_10_ IU/mL (median, 10–90% CI)	4.29 (2.51–5.26)	3.94 (1.68–4.47)	0.02
HBsAg to HBV DNA ratio (median, 10–90% CI)	0.83 (0.54–2.56)	0.63 (0.46–1.32)	0.01

This retrospective study was conducted in accordance with the guidelines of the Declaration of Helsinki, the principles of Good Clinical Practice and according to standards of the local ethics committees. The ethical committee of Hannover Medical School approved this research project and waived the need for written informed consent because routine diagnostic data have been analyzed anonymously.

### Serum HBsAg Quantification

Serum HBsAg levels were retrospectively quantified from serum samples stored at −20°C. Serum HBsAg levels were quantified using the Abbott ARCHITECT® assay (Abbott Diagnostics, Abbott Park, IL). The test has a dynamic range of 0.05–250 IU/ml. Samples were diluted 1∶100 in horse serum and if results were >250 IU/ml, samples were retested at a higher dilution. Samples with HBsAg levels <0.05 IU/ml were retested undiluted. Results are given in IU/ml. The inter- and intra-assay variability is approximately 10% (6.7–8.8%), according to the manufacturer datasheet. In addition, 26 HIV/HBV patient samples were also tested with the Roche Elecsys assay (Roche Diagnostics GmbH, Mannheim). The correlation of both assays was excellent (r = 0.99) (Figure S1).

### HBV-DNA, HIV-RNA and T-cells Measurement

HBV-DNA was quantified with COBAS AmpliPrep/COBAS TaqMan (Roche Diagnostics, Mannheim, Germany) with a lower limit of detection (LLOD) of 12 IU/mL in Hannover, Vienna, Duesseldorf and Bialystok. In Bonn and Berlin HBV DNA was quantified with the Abbott RealTime assay which has a LLOD of 10 IU/ml. HIV RNA was measured COBAS TaqMan HIV-1 Test (Roche Diagnostics, Mannheim, Germany) with LLOD of 50 copies/mL (Hannover, Vienna, Duesseldorf, Bialystok) and by Abbott Real-time HIV-1 test with LLOD 40 copies/mL (Bonn and Berlin). CD4, CD8 and CD3 T-lymphocyte counts were evaluated by flow cytometer Navios in Hannover and Duesseldorf (Beckman Coulter, Krefeld, Germany), FACS Calibur in Vienna, Berlin and Bialystok (Becton Dickinson, Heidelberg, Germany or Schwechat, Austria).

### Statistical Analyses

Serum HBsAg, HBV DNA and HIV RNA levels were logarithmically transformed. Data are presented as median (10–90% CI), unless indicated. Non-parametric (distribution-free) tests were applied. Mann–Whitney U and Kruskall–Wallis ANOVA tests were used for univariate and multivariate comparisons of independent continuous variables, Fisher’s exact test for discrete variables comparison. Multivariate analyses were performed by a stepwise linear regression with HBsAg levels as dependent value. The significance level (α) was assumed for P-value <0.05 for a two-sided tests. Statistical analyses were performed with Statistica 9.0 (Statsoft, Tulsa, USA) and GraphPad Prism 5.0 (GraphPad Software, Inc, La Jolla, USA).

## Results

HBsAg levels were quantified in 173 HIV/HBV co-infected patients (148 male, median age 40 yrs) recruited in 6 centers in Germany, Austria and Poland. In this large, multicenter, real-life setting cohort more than half of patients were HBeAg positive (n = 103, 59%). One hundred twenty three patients (72%) received cART active against HBV for a median duration of 47 (7–95) months. The detailed characteristics of the study cohort are presented in Table 1.

### HBsAg Levels in the Entire HIV/HBV Co-infected Cohort

**Figure 1:**
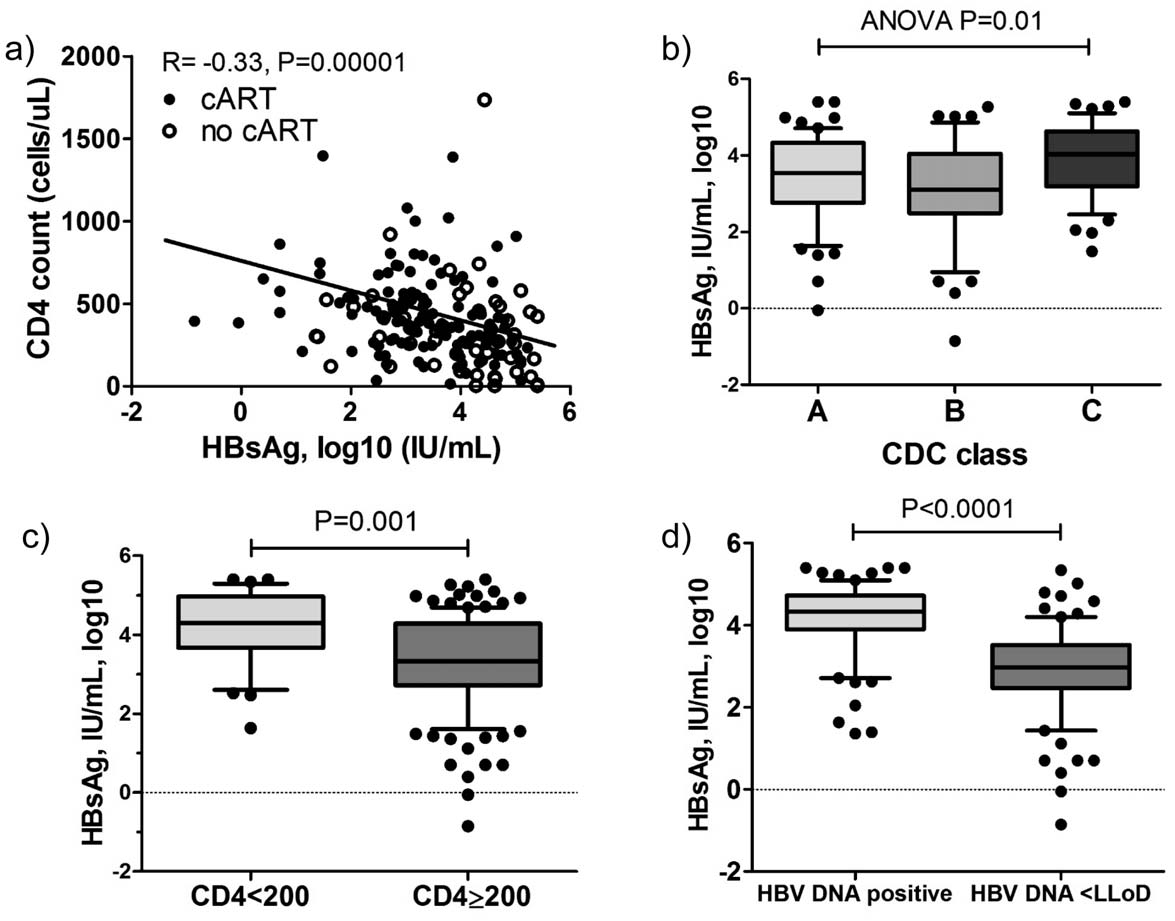
HBsAg levels in overall HIV/HBV cohort. a) Correlation of HBsAg levels with current CD4 T-cell count in all HIV/HBV co-infected patients. b) HBsAg levels in patients with different CDC stages. c) HBsAg levels in patients with CD4 T-cell counts <200 and ≥200/mm^3^ and d) in patients with undetectable HBV DNA (lower limit of quantification = LLOD) versus detectable HBV DNA. P-values obtained by Spearman correlation, U-Mann Whitney and Kruskall-Wallis ANOVA tests.

HBsAg levels showed a significant negative association with CD4 T-cell counts (R =  −0.33, P<0.001, Fig. 1a). HBsAg levels did not correlate with CD8 T-cell counts (r = −0.08, P = 0.27). Thus, a negative association between CD4/CD8 T-cells ratio and HBsAg levels (r = −0.29, P<0.001) was analogous to the one observed between CD4 T-cell counts and HBsAg. Furthermore, the highest HBsAg levels were observed in patients with most advanced stage of HIV-infection (CDC C: 4.03, CDC B: 3.11, CDC A: 3.53 log10 IU/mL, ANOVA P = 0.01, Fig. 1b). HIV/HBV co-infected patients with CD4 T-cell count below 200/mm^3^ had 1 log_10_ IU/mL higher HBsAg levels compared to patients with higher CD4 counts (4.30 vs 3.31 log_10_ IU/mL, P = 0.001, Fig. 1c). Nevertheless, HBV DNA remains the most important factor associated with HBsAg levels. Patients with undetectable HBV DNA had more than 1 log_10_ lower HBsAg level compared to patients with detectable HBV DNA (HBsAg 2.96 vs 4.33 log_10_ IU/mL, P<0.0001, Fig 1d).

### HBsAg Levels in HIV/HBV Co-infected Patients Treated with cART

**Figure 2:**
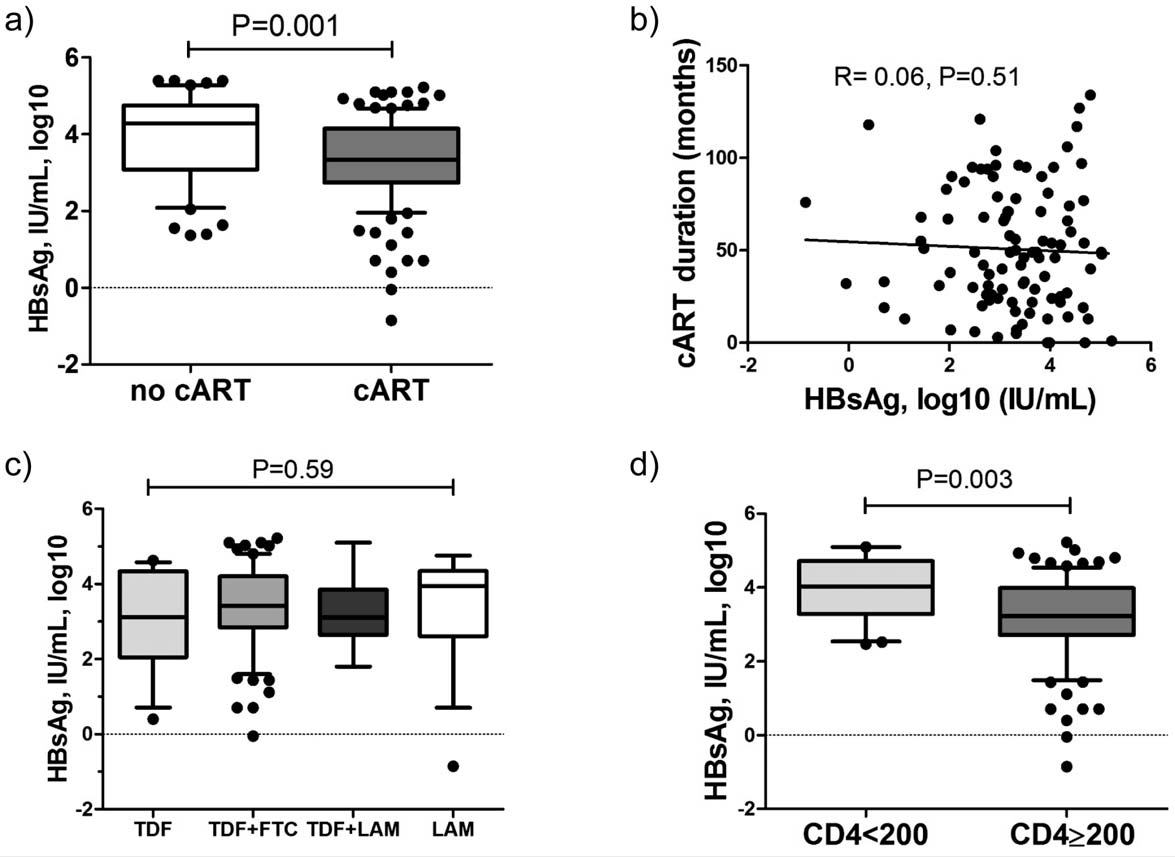
HBsAg levels in HIV/HBV patients receiving cART. a) Comparison of HBsAg levels in HIV/HBV co-infected patients receiving cART and not undergoing antiviral therapy. Lack of association between HBsAg levels and b) cART duration and c) cART regimen. d) HBsAg levels in patients receiving cART with current CD4 T-cell counts <200 and ≥200/mm^3^. P-values obtained by Spearman correlation, U-Mann Whitney and Kruskall-Wallis ANOVA tests.

123 HIV/HBV co-infected patients received cART active against HBV (Table 1). Median HBsAg levels in patients on therapy were lower by almost 1 log_10_ in comparison to untreated individuals (3.32 vs 4.23, P<0.001, Fig 2a). Comparing HBeAg positive and HBeAg negative patients, the difference in HBsAg levels between untreated and treated HIV/HBV co-infected patients was only significant in HBeAg positive patients (HBeAg positive 4.62 vs 3.71 IU/mL, P = 0.0003; HBeAg-negative 3.53 vs 3.02 IU/mL, P = 0.20). This suggests that anti-HBV therapy has more impact on HBsAg levels in HBeAg positive patients as also seen in HBV mono-infection [18].

HBsAg levels were higher in cART treated patients with HBeAg positive disease compared to HBeAg negative disease (4.62 vs 3.54, P = 0.002) and HBsAg was associated with HBV DNA (r = 0.56, P<0.001), while there was no correlation with ALT activity (r = 0.08, P = 0.34). Surprisingly, neither duration of anti-HBV therapy (r =  −0.06, P = 0.51, Fig 2b) nor the anti-HBV regimen (ANOVA P = 0.59, Fig 2c) was associated with HBsAg levels. On the other hand, again the stage of HIV-infection was associated with HBsAg levels, with highest levels observed in CDC-C category (A: 3.31, B: 3.03 and C: 3.77, P = 0.01).

In this large cohort of patients receiving cART an inverse correlation between HBsAg levels and CD4 T-cell counts was observed (r =  −0.34, P<0.001) (Table 3). Again, this association was much more pronounced in HBeAg positive patients (r =  −0.38, P = 0.001) as compared to HBeAg negative HBV-infection (r =  −0.31, P = 0.04). Of note, HBsAg levels were higher in patients with CD4 T-cells <200/mm^3^ (4.03 vs, 3.22 log_10_ IU/mL, P = 0.003, Fig. 2d).

**Table 3:** Univarite correlations and linear stepwise multivariate regression analysis of HBsAg levels and clinical and laboratory parameters in HIV/HBV co-infected patients receiving cART.

Serum HBsAg (IU/mL) vs	Univariate correlations		Multivariate regression
	R-value	P-value	P-value
Age (years)	−0.17	0.06	0.06
Therapy duration (months)	−0.06	0.52	
HBV DNA (IU/mL)	0.45	<0.001	<0.001
HIV RNA (cps/mL)	0.24	0.02	0.15
CD4 count (cells/µL)	−0.34	<0.001	0.06
Nadir CD4 count (cells/µL)	−0.06	0.57	
ΔCD4 (cells/µL)	−0.25	0.01	
CD8 count (cells/µL)	0.02	0.98	
ALT (IU/mL)	0.09	0.34	
Platelets (10^9^/mL)	−0.13	0.17	

In order to confirm these findings a multiple regression analysis with HBsAg levels as dependent value was performed in HIV/HBV co-infected patients receiving cART. HBsAg levels showed the strongest association with HBV DNA (β = 0.51, P<0.001). Among other factors with marginal significance CD4 T-cell count (β =  −0.21, P = 0.06) and age (β =  −0.20, P = 0.06) were noted (Table 3). When we exclude HBV DNA from this analysis (because 77% of patients had undetectable HBV DNA), CD4 T-cell count was the only significant factor independently associated with HBsAg levels (β =  −0.30, P = 0.006).

**Figure 3:**
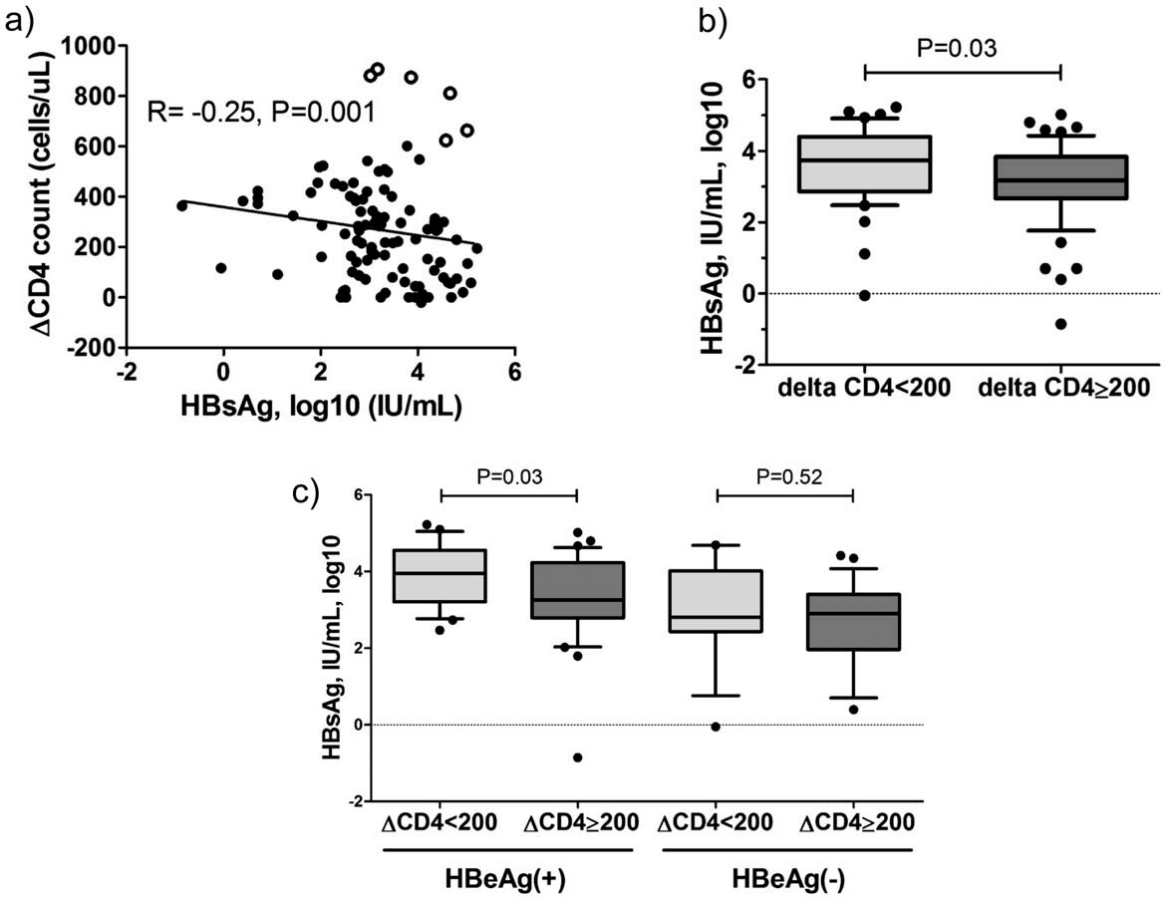
HBsAg levels and CD4 T-cell count dynamics. a) The correlation between CD4 T-cell count increase on cART (ΔCD4) and HBsAg levels (open circle denote outlier patients with delta CD4 T-cell >600/mm^3^ but high HBsAg concentrations. 5/6 HBeAg positive, 3/6 (50%) had initially very low nadir CD4 T-cell count (9, 38 and 95 cells/µL). b) Differences in HBsAg levels in HIV/HBV co-infected patients with ΔCD4 T-cell counts <200 and ≥200/mm^3^ in patients receiving cART and c) in HBeAg positive and HBeAg negative disease. P-values obtained by Spearman correlation and U-Mann Whitney tests.

Finally, to explain an association between HIV related immunodeficiency and HBsAg levels in more detail we have analyzed the effect of cART induced immune restoration, in this case a difference between current CD4 T-cell count and nadir CD4 T-cell count (ΔCD4). In patients receiving cART the median CD4 T-cell count increase was 166/mm^3^ (16–409) and was not different between HBeAg positive and HBeAg negative patients (158 vs 182 cells/µL, P = 0.61). ΔCD4 showed a correlation with the duration of cART therapy (R = 0.25, P = 0.02) and also with HBsAg levels (R =  −0.24, P = 0.02, Fig. 3a), suggesting a link between a restoration of immune functions and HBsAg levels. In patients receiving cART with ΔCD4>200/mm^3^ HBsAg levels were significantly lower compared to patients with ΔCD4<200/mm^3^ (3.31 vs 3.73 log10 IU/mL, P = 0.03, Fig. 3b). The distribution of HBeAg positive patients in the two groups was comparable (57% vs 62%, P = 0.59, respectively). The effect of immune reconstitution on HBsAg was more pronounced in HBeAg positive patients (Fig. 3c).

### HBsAg Levels in Untreated Patients with HIV/HBV Co-infection

**Figure 4:**
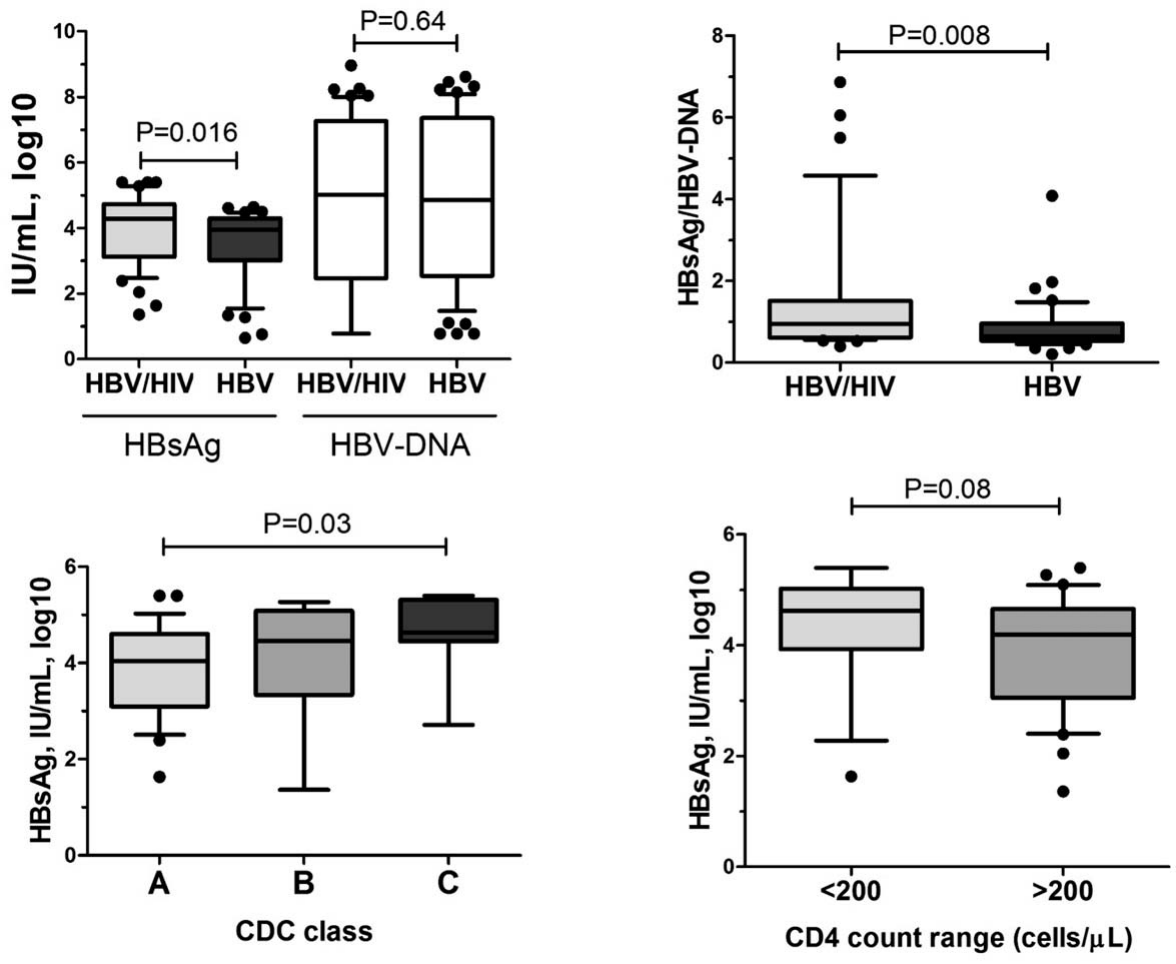
HBsAg levels in HIV/HBV patients not receiving cART. Comparison of HBsAg and HBV-DNA levels a) and HBsAg/HBVDNA b) in HIV/HBV co-infected patients not receiving cART with HBV mono-infected individuals without anti-HBV therapy matched for age, gender, HBeAg status and HBV DNA. HBsAg levels in HIV/HBV co-infected patients without cART in c) different CDC stages and d) in patients with CD4 T-cell counts <200 and ≥200/mm^3^. P-values obtained by U-Mann Whitney and Kruskall-Wallis ANOVA tests.

To assess a potential influence of HIV infection on HBsAg levels, we compared HBsAg levels in 46 HIV/HBV co-infected patients not receiving anti-HBV therapy with 46 HBV mono-infected untreated individuals matched for age, sex, HBeAg status and HBV DNA concentration, which are considered the most relevant factors associated with HBsAg concentrations [3], [7] (Table 2). HBsAg levels were higher in HIV/HBV co-infected patients vs. HBV mono-infected patients (4.29 vs 3.94 log10 IU/mL, P = 0.016) despite similar HBV DNA concentration (Fig. 4a). Thus, HBsAg productivity (HBsAg/HBV-DNA ratio) was significantly higher in patients with HIV/HBV co-infection (0.83 vs 0.63, P = 0.008, Fig. 4b).

Similar to previous reports in HBV mono-infected patients [7], [8], HBsAg levels were significantly higher in HBeAg positive HIV/HBV patients compared with HBeAg negative HIV/HBV patients (4.62 vs 3.54 log10 IU/mL, P<0.001). Moreover, HBsAg levels showed a negative association with age (r =  −0.28, P = 0.04) and a positive one with HBV-DNA (r = 0.37, P = 0.02). These correlations were less pronounced in HIV/HBV than in HBV mono-infected controls (r =  −0.49, P<0.001 and r = 0.57, P<0.001, respectively). Not only HBV related factors influenced HBsAg concentrations. Also in untreated patients, HBsAg levels were higher in patients with more advanced stages of HIV-infection (CDC-A: 3.98, B: 4.46 and C: 4.63 log10 IU/mL, ANOVA P = 0.03, Fig. 4c). Secondly, in this limited number of patients we observed a trend towards a negative association between HBsAg levels and current CD4 T-cell counts (r =  −0.24, P = 0.08). HBsAg levels tended to be higher in patients with CD4 T-cell counts below 200/mm^3^ (4.61 vs 4.19 log10 IU/mL, P = 0.08, Fig 4d).

## Discussion

In this retrospective multi-center study we provide data on HBsAg concentrations in a large cohort of HIV/HBV co-infected patients that further support the important role of the immune status for HBsAg decline.

In the overall cohort of 173 HIV/HBV patients we show a significant negative correlation between CD4 T-cell counts and HBsAg levels (Fig. 1a). We demonstrate that patients with less than 200/mm^3^ CD4 T-cells had significantly higher HBsAg levels compared to patients with higher CD4 T-cell counts (Fig. 1c) supporting the hypothesis that an intact immune status is important for inhibiting HBsAg production [6].

Combination antiretroviral therapy does not only restore CD4 T-cell counts but also inhibits HBV DNA, which both are relevant factors associated with HBsAg levels, especially in HBeAg positive disease [19] (Fig. 1d). Many studies have shown that HBV suppression by NA has only a limited effect on HBsAg decrease [5], [6], [18], [20] with approximately 0.15–0.81 log_10_ decline after 2 years during effective entecavir or tenofovir therapy [18]. The decline was documented to be higher in HBeAg positive patients than in HBeAg negative patients. In this study, patients on cART (median duration 3.9 years) had approximately 1 log_10_ lower HBsAg levels compared to untreated HIV/HBV co-infected patients (Fig. 2a). Considering that 60% of the HIV/HBV patients are HBeAg positive, the effect of NA therapy seems to be in the same range as in HBV mono-infected patients. We did not find a significant association between the NA regimen and HBsAg levels (Fig. 2c) and also HBV DNA (data not shown). This is in agreement with our data in HBV mono-infected patients where we did not observe a difference between potent NA (tenofovir, entecavir) and less potent NA (lamivudine, adefovir) in HBsAg decrease [6]. We also did not observe a correlation between treatment duration and HBsAg level. This could suggest that some patients may have experienced a stronger HBsAg decline and others not at all. In HBV mono-infected patients we have shown that approximately 1/5^th^ of patients experienced a pronounced HBsAg decline, while 28% had no decline at all during up to 9 years treatment [6]. The recent study by Thibault also confirmed that only 39% of HIV/HBV co-infected patients achieved any HBsAg decline during treatment containing tenofovir [16].

Most data suggest that direct targeting the HBV polymerase has limited impact on HBsAg levels. Nevertheless, HBV DNA suppression is important for HBsAg reduction. Also in HIV/HBV patients on cART, HBV DNA levels were the strongest factor associated with HBsAg concentrations (Table 3). After complete suppression of HBV DNA replication, some level of immune response is required to achieve further reduction of HBsAg or even HBsAg loss. In HIV co-infected patients, cART including HBV polymerase inhibitors can restore CD4 immune responses that may explain the impact on HBsAg levels in HIV/HBV patients. Importantly, the difference of nadir CD4 T-cell count and CD4 T-cell count at the time of HBsAg quantification (ΔCD4) was significantly correlated with HBsAg concentrations in patients on treatment (Fig. 3a). For example, patients with a ΔCD4 count of >200/mm^3^ had significant lower HBsAg levels than patients with ΔCD4<200/mm^3^ (Fig. 4b). Our data suggest that the effect of ΔCD4 T-cell count on HBsAg levels may be stronger in HBeAg positive patients than in HBeAg negative patients (Fig. 3c). Patients with HBeAg positive wildtype HBV infection are in an earlier stage of infection. The immune status may have more impact during earlier stages of HBV infection compared to later stages of HBV infection where T cell responses against HBV might already be exhausted [21].

The most convincing data for the important influence of the immune status on HBsAg levels is given by our data in untreated HIV/HBV co-infected patients. Here we show for the first time that HBsAg levels and HBsAg production were significantly higher in untreated HIV/HBV co-infected patients compared to a well-matched control cohort of HBV mono-infected patients (Fig. 4). Importantly, both groups had similar levels of HBV DNA, which was the strongest factor associated with HBsAg (Table 3). Also in this cohort, the highest HBsAg concentrations were observed in patients with more advanced HIV disease (Fig. 1c). Older studies published before the era of potent antiretroviral/anti-HBV treatment showed that chronic hepatitis B in co-infected patients takes a more progressive disease compared to mono-infected patients [11]. The higher HBsAg concentrations in HIV/HBV co-infected patients may be one reason for this observation as high HBsAg levels have recently been associated with disease progression independent of HBV DNA levels [22]. However, a limitation of this study is that we cannot correlate HBsAg levels with the stage of liver disease, as liver biopsies have not been performed in the majority of patients.

In conclusion our data support the hypothesis that significant HBsAg decline requires both, HBV DNA suppression and an effective immune response. This study helps to explain why immunmodulatory therapies such as IFN are more effective in reducing HBsAg levels than NA alone [5] supporting the demand for new immunomodulatory treatment concepts to increase the rates of HBsAg clearance. Our results also support the recommendations to initiate cART early in HBV/HIV co-infected patients. Restoration of the immune response in an earlier phase of HBV infection may be important to achieve significant HBsAg decline during cART including HBV polymerase inhibitors. The study has limitations due to its cross-sectional approach. HBV mutations, adherence to therapy and HBV genotypes were not considered, which may have influenced HBsAg levels. Prospective longitudinal data are necessary to validate whether CD4 immune reconstitution has an impact on HBsAg decline and which specific immunologic pathways are involved.

## Supporting Information

Figure S1 **Correlation of serum HBsAg levels measured by Architect Abbott and Elecsys Roche systems in 26 HBV/HIV infected patients.** P-value obtained by Spearman correlation test.
(TIF)
