# Supplementary figures and images for: Hepatitis B Surface Antigen Concentrations in Patients with HIV/HBV Co-Infection
Source: PLoS One. 2012 Aug 15;7(8):e43143. doi: 10.1371/journal.pone.0043143 (PMC3419648; doi:10.1371/journal.pone.0043143)

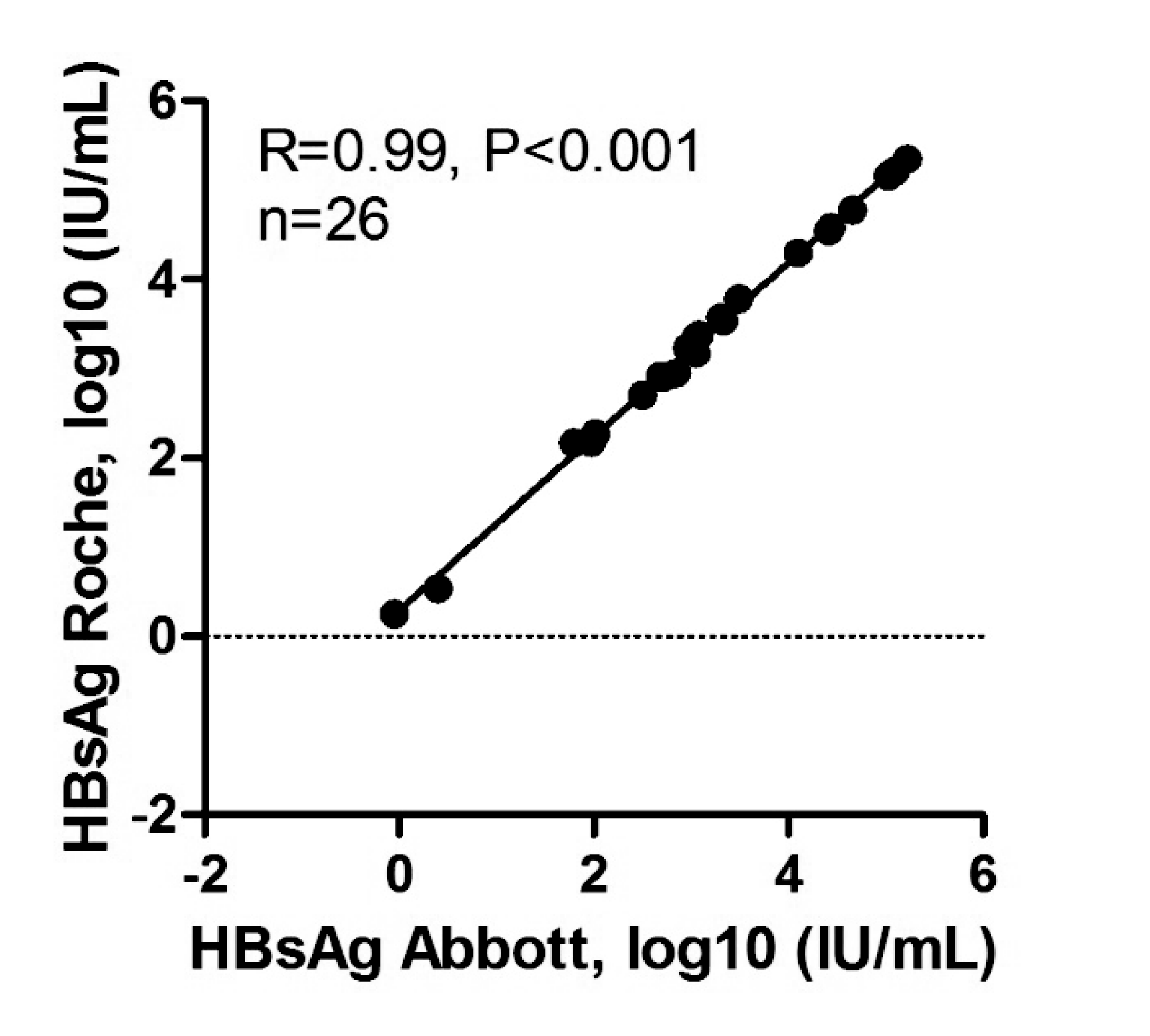

Supplement: Figure S1 — Correlation of serum HBsAg levels measured by Architect Abbott and Elecsys Roche systems in 26 HBV/HIV infected patients. P-value obtained by Spearman correlation test. (TIF) [file pone.0043143.s001.tif]
